# Clinical impact of coronal-STIR sequence in a routine lumbar spine MR imaging protocol to investigate low back pain

**DOI:** 10.1097/MD.0000000000010789

**Published:** 2018-06-18

**Authors:** Valeria Romeo, Carlo Cavaliere, Carmine Sorrentino, Andrea Ponsiglione, Lorenzo Ugga, Luigi Barbuto, Francesco Verde, Mario Covello

**Affiliations:** a Department of Advanced Biomedical Sciences, University of Naples Federico II; b IRCCS SDN, Naples, Italy.

**Keywords:** coronal-STIR MR sequence, extraspinal MR imaging findings, low back pain, lumbar spine MR imaging protocol

## Abstract

Aim of this study is to assess the clinical impact of coronal short tau inversion recovery (STIR)-weighted magnetic resonance (MR) sequence, when acquired in a lumbar spine MR imaging protocol, in detecting significant extraspinal imaging findings in patients with low back pain (LBP).

We retrospectively evaluated 931 lumbar spine MR examinations of patients with LBP. Extraspinal MR imaging findings were categorized as: probably related to LBP (Category 1), not related to LBP but with relevant implications on patient's care (Category 2), and not related to LBP without significant implications on patient's care (Category 3). For each MR imaging finding was also assessed if it was detectable or not on the conventional sagittal and axial acquisition planes.

Of the 931 evaluated MR examinations, 60 (6.4%) showed additional extraspinal MR imaging findings, categorized as follows: 55% (33/60) probably related to LBP (Category 1), 22% (13/60) not related to LBP but with relevant implications on patient's care (Category 2), and 23% (14/60) not related to LBP and without significant implications on patient's care (Category 3). Among categories 1 and 2 (n = 46), the 72% (33/46) of imaging findings were detected only on coronal plane. Coronal-STIR sequence significantly changed patients’ diagnostic work-flow in 3.5% (33/931) of cases.

Coronal STIR sequence, acquired in a lumbar spine MR imaging protocol to investigate LBP, may aid radiologists in detecting additional extraspinal MR imaging findings that could be related to LBP, addressing to the most appropriate clinical management.

## 1 Introduction

Low back pain (LBP) is defined as pain, muscle tension or stiffness localized below the costal margin and above the inferior gluteal folds, with or without leg pain (sciatica).^[1]^ Nonspecific LBP is usually classified according to duration as acute (less than 6 weeks), subacute (between 6 weeks and 3 months), or chronic (longer than 3 months).^[2]^ LBP is a very common condition that up to 84% of adults will experience during their lives, and up to 50% of them will have more than one episode and it is the second ranked cause of lost days at work.^[3]^ The major sources of LBP are, in descending order, spine, sacroiliac joint and hip, muscles, ligaments, and nerves.^[3,4]^ Spinal and extraspinal causes of chronic LBP are resumed in Table 1.^[5]^ After clinical evaluation, patients with LBP often undergo magnetic resonance (MR) of the spine as first level examination in order to assess the presence of spinal abnormalities, such as intervertebral disc protrusions, extrusions and any other sign of degenerative disease, as MR is considered the most appropriate imaging modality to investigate LBP.^[6,7]^ According to the American College of Radiology, pulse sequences commonly used in MR imaging of the spine are: 2-dimensional T1-weighted (T1w) sagittal imaging, 2-dimensional T2-weighted (T2w) or T2^∗^ sagittal imaging, 2-dimensional T1w axial imaging, 2-dimensional T2w or T2^∗^ axial imaging^[8]^; in addition, short tau inversion recovery (STIR) sequences are often performed to increase conspicuity of osseous and ligamentous lesions. Turbo spin-echo (TSE) T2w axial sequences are performed at selected spinal levels in case of disc protrusion, disc extrusion or in case of any abnormal finding detected on sagittal plane.^[9]^ Optionally, nonroutine MR sequences including diffusion weighted imaging, MR spectroscopy, in- and out-of-phase MR, and dynamic contrast-enhanced MR (perfusion imaging) can be acquired.^[8,10,11]^ The MR protocol of the lumbar spine performed at our institution to investigate subacute or chronic low-back pain includes T1w and T2w sagittal sequences, T2w sequence on axial plane conducted at selected spinal levels in case of disc protrusion or extrusion and STIR sequence on coronal plane. On the basis of our experience, we aim to demonstrate that coronal-STIR sequence should be acquired in a lumbar spine MR imaging protocol to investigate LBP, in order to provide a panoramic abdominal and pelvic view and to allow the evaluation of sacroiliac joints, coxofemoral joints, and abdominopelvic organs that, where affected, could be strongly related to LBP.

**Table 1:**
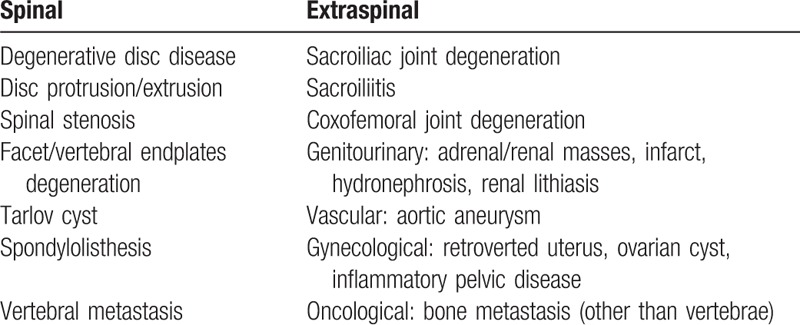
Spinal and extraspinal causes of LBP.

## 2 Materials and methods

### 2.1 Patient population and study design

**Figure 1:**
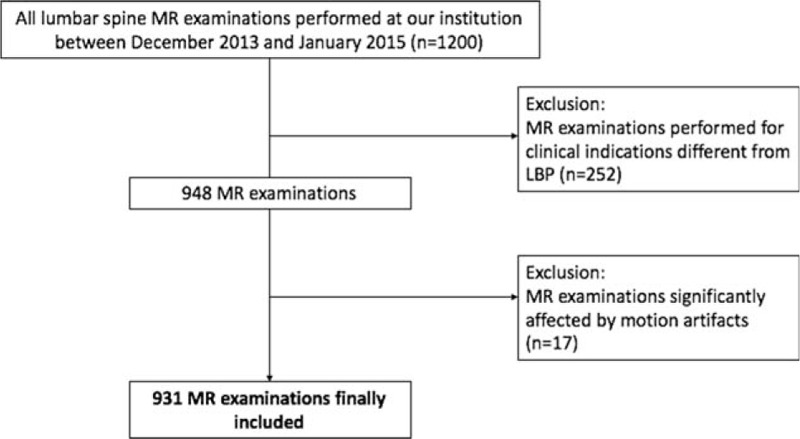
Flow diagram showing patient selection.

This retrospective, observational study followed the “Strengthening the Reporting of Observational Studies in Epidemiology (STROBE) Statement” and was approved by our local Institutional Review Board. All patients gave written informed consent to the execution of the MR examination. Between December 2013 to January 2015, 1200 consecutive patients (756 M, mean age, 62 years; age range, 30–87 years) underwent MR examination of the lumbar spine at our institution. Inclusion criteria were as follows: patients with subacute or chronic LBP with clinical indication to perform an MR examination of the lumbar spine. Exclusion criteria were: MR examination performed for clinical indications different from LBP (e.g., post-surgical follow-up, acute LBP, congenital diseases); MR studies significantly affected by motion artifacts, as evaluated by a radiologist not involved in the subsequent image analysis; and MR examinations stopped before the acquisition ended due to claustrophobia. Of the 1200 patients initially selected, 931 were included in the study. The flow chart of patient selection is reported in Fig. 1.

### 2.2 MR imaging protocol

**Figure 2:**
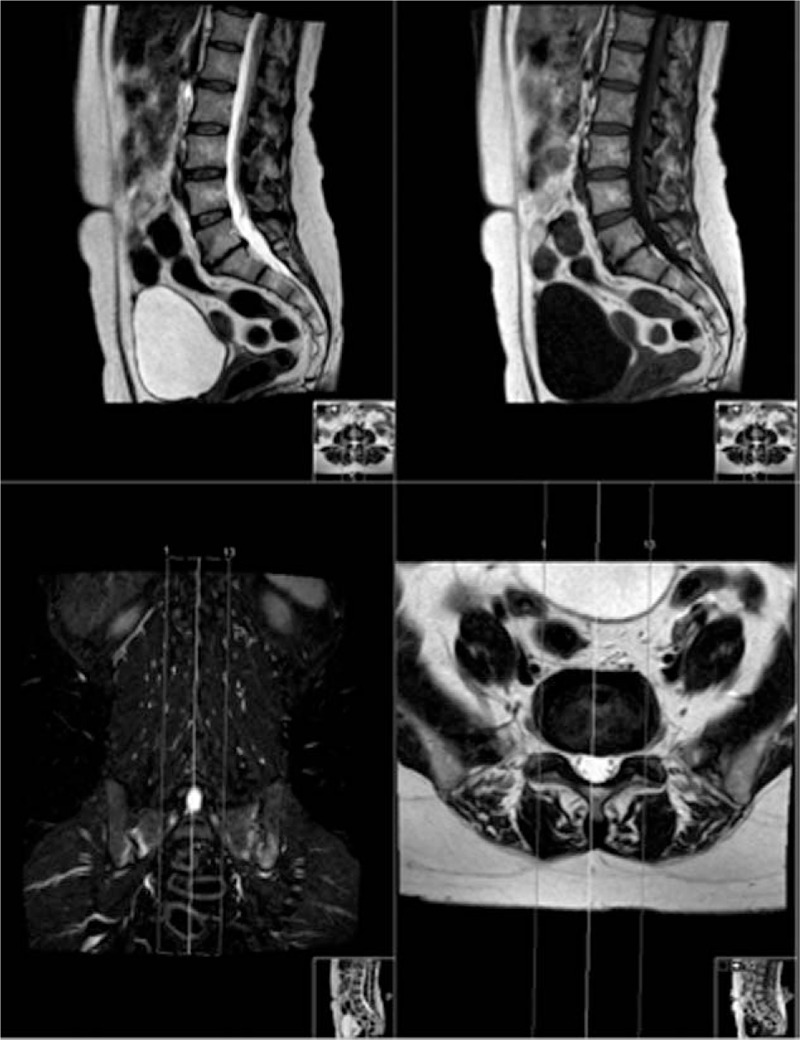
FOV positioning for obtaining a sagittal sequence. Of note, sacroiliac/coxofemoral joints and renal lodges are not included.

Patients underwent MR imaging on different MR scanners sited at our Institution (Achieva 1.5T, Philips Healthcare, Best, Netherlands; Signa Excite HD 1.5T and Optima 1.5T, GE Healthcare, Milwaukee, WI; Panorama HFO 1.0T, Philips Healthcare, Best, Netherlands) using a phased array surface coil. Sagittal T1-w, sagittal T2-w, and coronal-STIR images were obtained. Axial T2w sequence was performed on selected spine levels in case of disc abnormalities detected on sagittal planes; if no abnormalities were detected on sagittal planes, T2w sequence was acquired conventionally from the third lumbar to the first sacral vertebra. All acquisition parameters for each MR scanner are summarized in Table 2. The coronal plane and the field of view (FOV) of the STIR sequence included the entire abdomen and were angulated in order to include coxofemoral joints and the sacrum, as shown in Fig. 2.

**Table 2:**
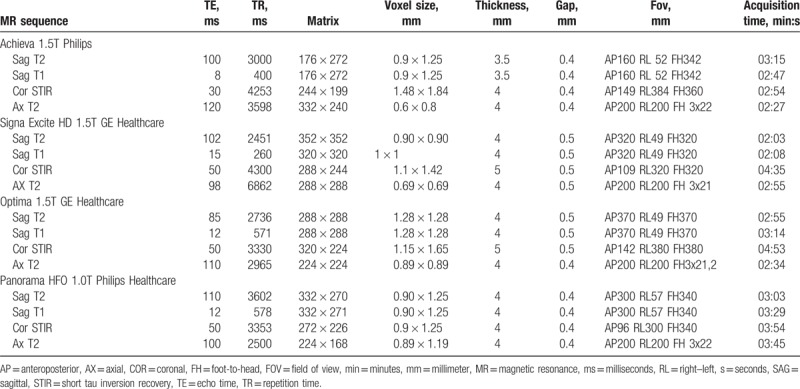
MR sequence parameters for each MR scanner.

### 2.3 Image analysis

Two radiologists with respectively 20 and 6 years of experience in Neuroradiology analyzed both standard and STIR images in consensus. A preliminary evaluation was made on the basis of T1-w and T2w sagittal images as well as on T2w axial images and a diagnostic hypothesis was formulated. Imaging findings were then integrated with coronal-STIR sequence and a final diagnosis was recorded. For spine evaluation, the presence of spinal curve abnormalities, scoliosis, degenerative disc disease, disc protrusion or extrusion, spinal stenosis, spondyloarthrosis, facet/vertebral endplates degeneration, and vertebral bone lesions was assessed. Extraspinal MR imaging findings, including sacroiliac and coxofemoral joints degeneration or sacroiliitis, vascular, genitourinary and gynecological diseases, were so categorized:

1. MR imaging findings probably related to LBP (Category 1);
2. MR imaging findings not related to LBP, with relevant implications on patient's care and management (Category 2);
3. MR imaging findings not related to LBP, without significant implications on patient's care and management (Category 3).

For each MR imaging finding was also assessed if it was included on conventional acquisition planes (sagittal and/or axial) or if it was detectable only on the coronal plane. For the most relevant extraspinal imaging findings, it was also determined whether the patients were aware of the emerged pathological condition. Clinically relevant imaging findings (Category 1 and 2) have been confirmed during the clinical and instrumental follow-up and/or after symptoms’ resolution following specific treatment.

## 3 Results

All spinal abnormalities detected on conventional sagittal T1w and T2w images were identified on coronal-STIR images.

Among the 931 evaluated MR examinations, 60 (6.4%) showed additional extraspinal MR imaging findings; of these, 68% (41/60) were detected only on coronal plane. A summary of extraspinal imaging findings is reported in Table 3. The 55% (33/60) of extraspinal imaging findings were considered probably related to LBP with relevant implications on patient's care (Category 1), 22% (13/60) were considered not related to LBP but with relevant implications on patient's care (Category 2) and 23% (14/60) were considered not related to LBP and without significant implications on patient's care (Category 3). Of Category 1 and 2 imaging findings (n = 46), 72% (33/46) were detected only on coronal plane. Final diagnosis of these findings is reported in Table 4 and for each imaging finding is also reported if the patient was aware or not about the emerged condition.

**Table 3:**
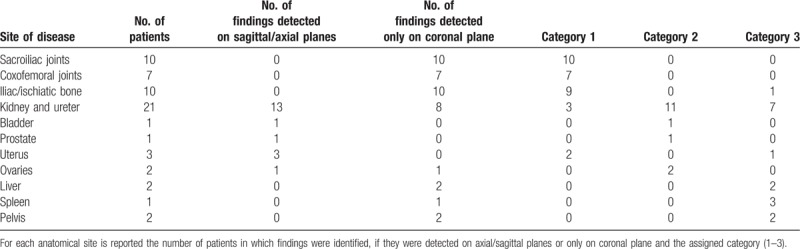
Summary of MR extraspinal imaging findings.

**Table 4:**
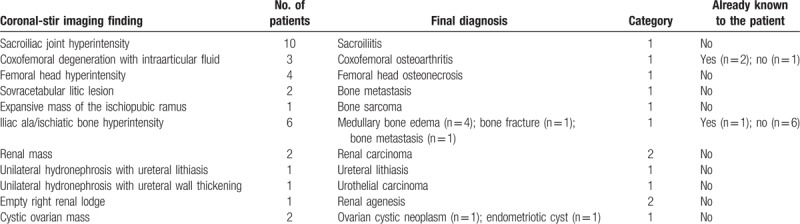
Final diagnosis of Category 1 and 2 extraspinal MR imaging findings (n = 33) detected only on coronal-STIR sequence.

**Figure 3:**
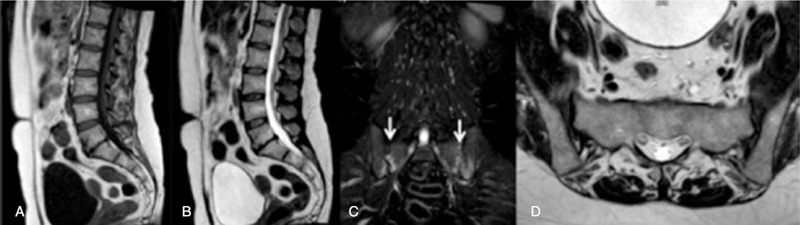
A 51-year-old female patient with chronic low back pain. (A) Sagittal T1-weighted sequence. (B) Sagittal T2-weighted sequence. (C) Coronal-STIR sequence. (D) Axial T2-weighed sequence. Conventional MR sequences acquired on sagittal plane showed a mild disc protrusion at the level of L5–S1 intersomatic space (A and B). On coronal-STIR sequence (C) a bilateral signal hyperintensity at the level of sacroiliac joints was detected (arrows); this finding is consistent with bilateral sacroiliitis and it was not detectable on axial plane (D).

**Figure 4:**
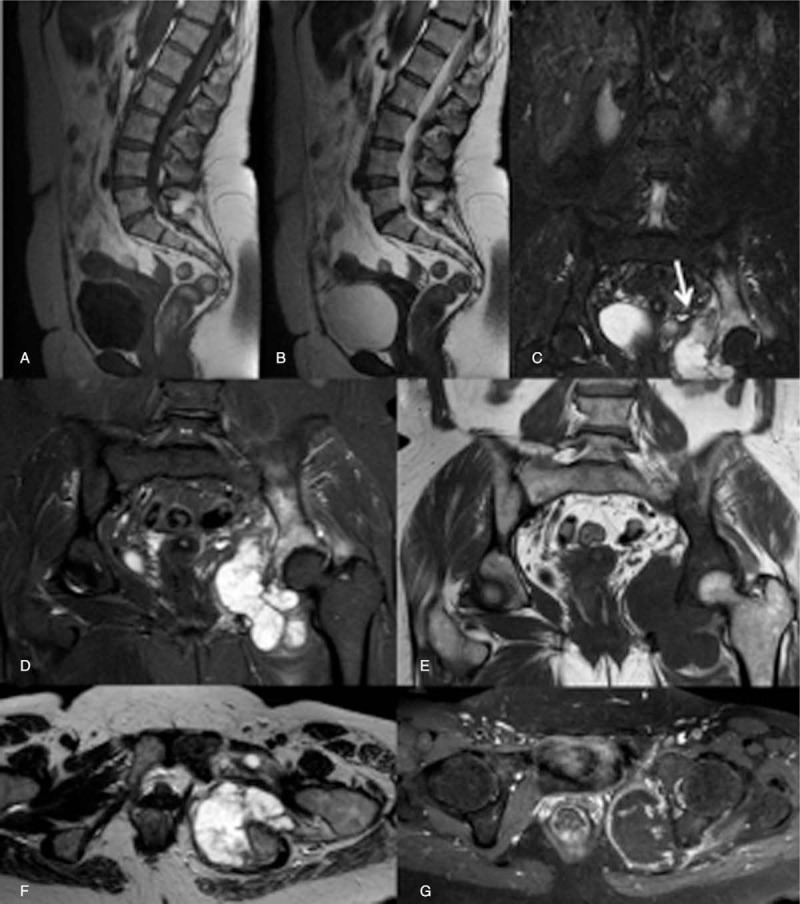
A 52-year-old female patient with chronic low back pain. (A) Sagittal T1-weighted sequence. (B) Sagittal T2-weighted sequence. (C and D) Coronal-STIR sequence. (E) Coronal T2-weighed sequence. (F) Axial T2-weighted sequence. (G) Axial T1-weighted 3D fat-sat MR sequence after contrast agent injection. Conventional MR sequences acquired on sagittal plane showed the presence of a mild disc extrusion at the level of the L5–S1 intersomatic space and a disc protrusion at the level of the L2–L3 intersomatic space (A and B). On coronal-STIR (C), a large expansive mass of the ischiopubic ramus with diffuse signal hyperintensity of the iliac bone was appreciated (arrow). The mass would have been missed on sagittal plane. (D–G) A following MR examination of the ischiopubic region confirmed the presence of a large expansive mass with irregular and peripheral enhancement, suggestive of a bone sarcoma.

**Figure 5:**
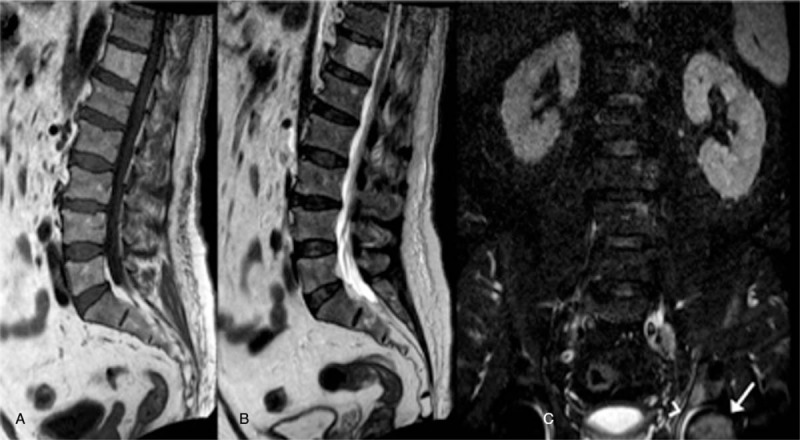
A 63-year-old male patient with chronic low back pain. (A) Sagittal T1-weighted sequence. (B) Sagittal T2-weighted sequence. (C) Coronal-STIR sequence. Conventional MR sequences acquired on sagittal plane showed the presence of spondylosis, multiple disc protrusions, and a large osteoangiomas of the T12 vertebral body. On coronal-STIR sequence (C) a signal hyperintensity of the left femoral head (arrow) and the acetabulum with intraarticular fluid (arrowhead) was detected; this finding was proved to be a femoral head osteonecrosis.

The majority of extraspinal MR imaging findings were renal in 30% of patients (18/60); of these, 14 were also detected on sagittal/axial planes while 4, belonging to Category 1 and 2 (3 exophytic renal masses, 1 case of polycystic kidneys with bilateral hydronephrosis and 1 case of unilateral hydronephrosis with underlying ureteral lithiasis), were not included on conventional acquisition planes and detectable only on coronal images. After renal disease, the most frequent extraspinal MR imaging findings detected on coronal-STIR sequence concerned sacroiliac joints in 17% (10/60) of cases (9 bilateral sacroiliitis and 1 unilateral sacroiliitis with associated sacrum hyperintensity on STIR sequence due to a bone abscess), ischiatic and/or iliac bone in 17% (10/60) of cases (6 benign bone lesions, 1 fracture, 2 bone metastasis, and 1 sarcoma), followed by coxofemoral disease in 12% (7/60) of cases (2 coxofemoral joints degeneration and 5 femoral head osteonecrosis); some examples are shown in Figs. 3–5: none of these imaging findings was detected on conventional sagittal and/or axial acquisition planes and all were considered probably related to LBP (Category 1). Ureteral MR imaging findings were found in 5% of cases (3/60) cases, all detected only on coronal plane and belonging to Category 2 and 3 (2 cases of ureteral wall thickening and one case of ureteral kinking). Gynecological extraspinal MR imaging findings consisted of 2 large uterine fibromas and 1 retroverted uterus with fibromatosis, followed by ovarian disease in 2 cases, 1 hemorrhagic cyst, and 1 ovarian cystic expansive mass. All findings were detected on sagittal/axial planes while the hemorrhagic ovarian cyst was detectable only on coronal plane. The 2 detected large uterine fibromas were considered probably related to LBP in 2 patients without any significant spinal abnormalities (Category 1); the large ovarian cystic mass was considered probably related to LBP (Category 1) even if in association with spondylosis, disc protrusions and end-plate degeneration, while the hemorrhagic ovarian cyst was considered not related to LBP but with relevant implication on patient's care (Category 2). Prostate gland enlargement due to an infiltrating tumor lesion was found in 1 patient (1.7%) on all acquisition planes and considered, in absence of vertebral metastases, not related to patient LBP but with relevant clinical implications (Category 2). An irregular vesical wall thickening with bilateral hydronephrosis (Category 2) was found in 1 case (1.7%) on all acquisition planes. Results of the retrospective analysis were concordant with that of the first MR evaluation. Category 1 and 2 imaging findings were confirmed during clinical and instrumental follow-up and/or after symptoms’ resolution following specific treatment. Coronal-STIR sequence also revealed the presence of other extraspinal imaging findings, such as renal agenesis, hepatic hemangioma, and a mild splenomegaly that, even if not related to LBP, were clinically relevant.

## 4 Discussion

On the basis of our results, the use of coronal-STIR sequence enabled radiologists to identify relevant MR imaging findings that would have been missed on sagittal or axial planes and that significantly changed patient's diagnostic work flow and prognosis in 3.5% (33/931) of cases, suggesting the most appropriate clinical management. Thanks to the additional scanning plane, the use of coronal-STIR sequence in our lumbar spine MR imaging protocol allowed the assessment of musculoskeletal and abdominopelvic imaging findings related to LBP and not included on sagittal and axial planes, providing exhaustive information regarding the vertebral column at the same time. The major extraspinal causes of LBP, affecting sacroiliac joints, coxofemoral joints or pelvic bones, are not routinely imaged by the conventional MR imaging protocol of the lumbar spine; furthermore, sacroiliitis, one of the most important causes of unresolved LBP, is better assessed on STIR images and detectable only on the coronal plane.^[12]^ Of the 46 relevant extraspinal imaging findings (Category 1 and 2) detected in our study, 71% (33/46) were not included on sagittal plane and would not have been identified using a conventional MR acquisition protocol of the lumbar spine. The pathological extraspinal imaging findings detected in our study were renal in 30% (18/60) of cases and 3 of these were considered related to LBP (2 patients with bilateral hydronephrosis and 1 patient with ureteral lithiasis and unilateral hydronephrosis). Even if several renal imaging findings were also detectable on axial planes, the coronal plane allowed their easier detection and characterization. After renal diseases, unilateral or bilateral sacroiliitis, coxofemoral disease and iliac/ischiatic bone lesions were the most frequent pathological extraspinal MR imaging findings in our study and were all considered related to LBP. LBP and sciatica were determined by an expansive tumor mass of the ischiopubic ramus in a patient who only showed a disc protrusion: this relevant finding was missed at sagittal and axial plane evaluation. Regarding gynecological findings it is reported that pelvic masses could directly compress the sacral nerves causing LBP or sciatica,^[13]^ as occurred in our study in 2 patients without spinal abnormalities and with respectively a large ovarian cystic mass and multiple uterine fibromas. Another relevant case we had in our study was the detection of an irregular enlargement of the prostate gland with multiple locoregional lymphadenopathies, which was proved to be an advanced prostate cancer (Category 2), unknown to the patient. Both uterus and prostate gland are commonly evaluated on sagittal plane; however, the lumbar spine MR imaging is not focused on pelvic region and the additional view provided by the coronal plane can help radiologists to advance a more precise diagnostic hypothesis. Previously, Gleeson et al^[14]^ determined the clinical impact of coronal-STIR sequence in addition to routine images of the lumbar spine in the evaluation of sacroiliac joints and sacrum obtained in patients referred for acute LBP; they concluded that routine coronal-STIR imaging of the sacrum improved diagnostic evaluation in a small number of cases (2%). More recently, Gupta et al^[15]^ have also assessed the additional merit of coronal-STIR sequence for MR imaging of lumbar spine in a smaller patient population; they retrospectively evaluated extraspinal findings (inflammatory sacroiliitis, sacroiliac joints degeneration, sacral stress fracture, muscular sprain, and atypical appendicitis). Similar to our results, they concluded that coronal-STIR imaging can provide additional diagnosis in a percentage of patients (6.8%) and that it should be included in the routine protocol for MR imaging of the lumbar spine. A recent paper investigated the role of an extended field-of-view lumbar spine MR in identifying incidental abdominopelvic findings, concluding that most incidental findings could be confidently dismissed without additional testing.^[16]^

Several authors reported many examples of extraspinal incidental findings detected on a conventional lumbar spine MR imaging (using only axial and sagittal planes), including vascular, genitourinary, gastrointestinal, musculoskeletal, and oncological findings^[17–23]^ but without including sacroiliitis and coxofemoral disease, which are the main extraspinal causes of LBP and commonly undetectable on sagittal and axial acquisition planes; moreover, since axial MR images are commonly acquired in order to define vertebral suspicious or abnormal findings detected on sagittal planes, the possibility to include additional extraspinal findings depends on the selected spinal level: this is the case of renal disease, which could be missed if the interested axial plane does not coincide with renal lodges. In addition, axial sequences are commonly acquired to evaluate disc protrusions or disc extrusions: for this reason, they are not often conducted below the first sacral vertebra.

Possible limitation of the use of coronal-STIR sequence could be the difficult evaluation of signal abnormalities of the anterior wall of the vertebral body, that is, the anterosuperior corner: since this site is most commonly involved after a spinal traumatic injury,^[24]^ in case of patients with a history of spinal trauma, additional sequences should also be performed, such as sagittal STIR sequence and axial TSE T1-w sequence; nevertheless, the traumatic involvement of the anterosuperior corner could be identified with sagittal T1w images. Involvement of the anterior corners can also occur in patients with negative spondyloarthritis^[25]^ and detected with our MR protocol on sagittal TSE T1w sequence with the combined possibility to assess sacroiliac joint involvement, which is a common finding in these patients, on coronal-STIR images. In addition, the employment of coronal-STIR sequence could increase the risk of overdiagnosis and the amount of additional and not really required examinations. In conclusion, a coronal-STIR-weighted MR sequence, included in lumbar spine MR imaging protocol to investigate LBP, may aid radiologists in detecting additional extraspinal MR imaging findings that could be related to LBP, addressing to the most appropriate clinical management.

## Author contributions

**Conceptualization:** Valeria Romeo, Carlo Cavaliere, Francesco Verde, Mario Covello.

**Formal analysis:** Valeria Romeo, Carlo Cavaliere, Lorenzo Ugga.

**Methodology:** Valeria Romeo, Carlo Cavaliere, Carmine Sorrentino, Luigi Barbuto, Mario Covello.

**Supervision:** Valeria Romeo, Carlo Cavaliere, Luigi Barbuto, Mario Covello.

**Validation:** Mario Covello.

**Writing – original draft:** Valeria Romeo, Carlo Cavaliere, Andrea Ponsiglione, Mario Covello.

**Writing – review & editing:** Valeria Romeo, Carlo Cavaliere, Andrea Ponsiglione, Lorenzo Ugga, Francesco Verde, Mario Covello.
